# Supplementary material for: A novel approach of creating sustainable urban planning solutions that optimise the local air quality and environmental equity in Helsinki, Finland: The CouSCOUS study protocol
Source: PLoS One. 2021 Dec 2;16(12):e0260009. doi: 10.1371/journal.pone.0260009 (PMC8638916; doi:10.1371/journal.pone.0260009)
Supplement: Attachment — Submitted filename: Response to reviewers.docx [file pone.0260009.s001.docx]

Reviewer #1: I have greatly enjoyed reading this study protocol on such an interesting multidisciplinary topic. I cannot wait to read the results and see the AI tool in action. My only recommendation is to add urban design experts to your research team to help with the urban design details and variables that could be included in the model to improve the results.

*Thank you for the positive feedback. We are working closely with the City of Helsinki, who are advising us on urban design and variables of interest.*

Reviewer #2: The paper is the protocol for a substantial and sophisticated study of air pollution and urban planning in one city. The paper is very well written, thoroughly grounded in existing literature, and detailed in explaining the aims, proposed data sources and methods for the study. I strongly support publication and have only two very minor comments.

*Thank you for the positive feedback.*

I was unclear how the study would deal with the increasing move to electric vehicles which have very different pollution profiles compared with those reliant on fossil fuels. There is a comment about vehicle fleet and emissions factors (p22, line334) but I didn’t see an explicit recognition of this. It would be helpful to have a sentence on how the study plans to cope with the uncertainties around rates of transition here.

*The reviewer is right that naturally the amount of electric vehicles will increase in future which will reduce traffic related pollutant emissions. However, there will likely be long transition period when both gasoline and electric vehicles will be present in the vehicle fleet. This reduction in can be taken into account in the emission factors like was made for example in our earlier study by Karttunen et al. (2020). In Northern latitudes road dust resuspended by traffic causes significant source for aerosols in spring time and as electric vehicles are heavier than gasoline cars (due to batteries) the road dust emissions are expected to increase in future. Also these can be taken into account in the emission factor inputs to the model (see Karttunen et al. 2020)*

*We have added the following sentence to the manuscript (paragraph starting at line 421):*

*“**The ongoing transition from gasoline vehicles to electric vehicles will be taken into account in the calculation of emission factors based on the predictions provided by traffic authorities (Karttunen et al. 2020). There will be significant amount of time when both vehicle types will co-exist during which particle emissions still take place. And even if all vehicles would be electric vehicles, in northern latitudes resuspended road dust causes significant source for particles in springtime. As electric vehicles are heavier than gasoline cars due to batterie) the road dust emissions are expected to increase in future. This will be included in the calculation of emission factors given to the model (Karttunen et al. 2020).”*

I was unclear how or whether the study will capture distributional effects or differences by socio-economic status. I think this is primarily through looking at where people live rather than how and when they move through urban spaces. There is a comment (p23, line356) that the study has data on socio-economic mobility profiles but the same paragraph states only that models will predict numbers of travellers on different modes, not broken down by socio-economic status. A sentence or two elaborating further how distributive impacts will be considered would be welcome.

*Thank you for this comment. We do not have any direct data on how people move, but we can model the changes over time, e.g. changes in socio-economic status, changes in neighbourhood composition or possibly household composition (only possible for densely populated areas). Different levels of movement will also be included in the traffic model.*

*We will clarify this in the text as follows (paragraph starting at line number 447):*

*“A DNNs method will be developed for generating the prediction of the amount of people in the traffic. For this, a number of socio-economic mobility profiles will be constructed based on micro-level socio-economic data and car ownership data, which will feed into training the learning algorithm, together with historical traffic data. The result will be a DNNs algorithm that has learned to predict the number of travellers of all transport profiles (vehicles, public transportation, pedestrian, bicyclist) passing the city area conditional on the weather, time of day, season, events, construction works and demographic structure at the area. Distributional effects of consequent predicted changes in air pollution will be examined to aid decision making on identifying optimal urban planning scenarios.”*
